# Systematic review of longitudinal studies on the association between cluster of health-related behaviors and tooth loss among adults

**DOI:** 10.1080/00016357.2023.2287718

**Published:** 2024-04-03

**Authors:** Fatimah Alobaidi, Ellie Heidari, Wael Sabbah

**Affiliations:** Faculty of Dentistry, Oral & Craniofacial Sciences, King’s College London, London, UK

**Keywords:** Adults, health behaviors, longitudinal studies, systematic review, tooth loss

## Abstract

**Objectives:**

To systematically review longitudinal studies on the association between cluster of/multiple health-related behaviors and tooth loss among adults.

**Materials and methods:**

Inclusion criteria were prospective and retrospective longitudinal studies; adults; multiple or cluster of behaviors; tooth loss, one or more tooth lost and complete tooth loss. Exclusion criteria were intervention studies; cross-sectional studies; case-control studies; children under 18years-old; single behavior. Two reviewers searched three databases up to April 2023. Open Grey and Google Scholar were searched for grey literature.

**Results:**

Twelve longitudinal studies were included in this review. Nine studies had good quality, two had poor quality, and one had fair quality according to New-Castle-Ottawa Scale. According to ROBINS-E tool, nine studies were judged as moderate risk of bias while two studies were at low risk of bias and one study had serious risk of bias. One study assessed cluster of behavior, while others examined a number of separate health-related behaviors in relation to tooth loss. Meta-analysis was not feasible because of the high heterogeneity in exposure, measure of outcomes, covariates, sample size, and follow-up time. The research found an association between tooth loss and oral hygiene practices (two studies), dental attendance (four studies), smoking (six studies), and alcohol consumption (three studies).

**Conclusion:**

This review provides evidence of a longitudinal association between cluster of/multiple health related-behaviors and tooth loss.

## Introduction

One of the important indicators of poor oral health is the number of teeth present in the oral cavity. Tooth loss can have a significant impact on quality of life, can lead to inadequate intake of essential nutrients, and discourage people from engaging in social events [1]. Accounting for the known behavioral factors that affect tooth loss and the common risk factors for oral health and general health have been the main focus of preventive oral health research [2]. There are multiple factors that contribute to tooth loss including oral conditions such as periodontal diseases and dental caries [3,4], and general conditions [5]. These factors can be increased by engaging in risk behaviors. Health-risk behaviors are defined as any harmful act that can increase the possibilities of diseases or delay healing [6]. Several studies have found a correlation between tooth loss and oral health behaviors such as toothbrushing, and frequency of dental visits [7]. Others have suggested that tooth loss has a strong link with other behavioral factors such as smoking [8,9] and consumption of alcoholic drinks [10,11]. However, other behavioral factors could have an indirect and less obvious association with tooth loss. Recent research findings reported an association between better oral health and physical activity, and argued that being active promotes a positive inflammatory response and potentially protects against tooth loss [12]. Other findings suggested a link between diet and oral health and reported that consumption of fruits and vegetables may halt periodontitis progression and may prevent tooth loss [13].

Risk behaviors frequently occur in groups and clusters rather than in isolation [14,15]. Engaging in multiple risk behaviors is more common among people with poorer oral health [16,17]. Exploring the effect of multiple health-related behaviors rather than single behavior provides a more comprehensive view of the factors that contribute to tooth loss. This is particularly important due to the complexity of the determinants of tooth loss which could include different factors such as diet, lifestyle, and overall health. For instance, researchers have found that people who smoke and have poor oral hygiene are at higher risk of tooth loss [18,19]. Developing an understanding of these combined risks and how health behaviors co-occur (different behaviors occurring among the same population) or cluster (different behaviors cluster together among certain population) is important to gain knowledge about the contributing effect of different health-related behaviors on tooth loss. Previous systematic reviews have assessed the relationship between oral health and individual behaviors such as smoking [18,20], beverage consumption [21,22], diet [13,23], and physical activity [24]. However, previous reviews mainly focused on one behavior at a time. Little is known about the effect of multiple behaviors on tooth loss among the same population. To our knowledge, there is no systematic review that assessed the longitudinal association between multiple health-related behaviors and tooth loss in the same population. Thus, the aim of this paper is to systematically review existing longitudinal studies on the association between cluster of/multiple health-related behaviors and tooth loss among adults.

## Materials and methods

This systematic review was conducted according to the Preferred Reporting Items for Systematic Reviews and Meta-analyses (PRISMA). The study protocol was listed in the International Prospective Register of Systematic Reviews (PROSPERO) (Registration number CRD42022367174).

### Criteria for study consideration

Eligibility was determined based on PRISMA 2020 guidelines, which include PECO, as ‘P,’ population ‘adults’ (aged 18 and over at baseline), ‘E,’ exposure ‘cluster of/multiple health-related behaviors,’ ‘C,’ comparison group ‘not exposed to multiple behaviors’, and ‘O,’ outcome ‘tooth loss’.

***Inclusion criteria:***

- Prospective and retrospective longitudinal studies.
- Participants aged 18 years-old and more at baseline.
- Exposure included cluster or multiple health-related behaviors.
- The outcome is tooth loss, one or more tooth lost, and complete tooth loss (clinically examined or self-reported).

***Exclusion criteria:***

- Randomized and non-randomized controlled trails.
- Intervention studies.
- Cross-sectional studies.
- Case-control studies.
- Studies which included participants younger than 18 years old at baseline.
- Studies with a single behavior.

### Study selection and data extraction

The literature review was conducted by two independent reviewers through three databases (MEDLINE *via* PubMed, EMBASE *via* Ovid and LILACS *via* BIREME) using Medical Subject Headings (MeSH) terms and text words around the main topics: the outcome (tooth loss) and exposure (cluster of health-related behaviors). The search in each data source was until April 2023. The three databases (MEDLINE, EMBASE, and LILACS) were deemed to be sufficient to identify relevant literature and consistent with the common recommendation for systematic reviews for searching biomedical literature. The articles were filtered for relevancy by their titles and by their abstracts before they were finally included by reading the full article. All relevant papers were referenced using Endnote X9.

### Search strategy

the following search terms were used to search all studies and databases, tooth loss OR edentulous OR absence of teeth OR tooth retention OR oral health OR functional dentition AND cluster of behaviour OR cluster of behavior OR health-related behaviour OR health-related behavior OR behaviour OR behavior OR risk factors OR risk bahviour OR risk behavior AND longitudinal OR prospective OR retrospective studies. Date restrictions were applied to papers published between 2000 to April 2023. English language restriction was also applied. The search strategy for each database is shown in (Appendix 1, supplementary material). We also searched reference list of published papers. Google Scholar and OpenGrey were used to look for unpublished relevant grey literature. Authors who are known to work in this area were contacted to identify missing and/or relevant data.

### Selection process

Two reviewers independently evaluated eligibility in a standardized, blinded manner. Following the PRISMA flow diagram, a flowchart was made that shows the number of studies at each stage of the evaluation and the reasons for exclusion after determining their eligibility. Reviewers’ disagreements were settled through discussion and by a decision of a third reviewer to reach an agreement.

### Data collection process

Two reviewers extracted data on the study design, authors, publication year, country, participants’ characteristics (sample size, age, follow-up duration), exposures including cluster or multiple health-related behaviors (oral hygiene practices, dental service utilization, smoking status, and alcohol intake), outcomes (complete tooth loss and/or partial tooth loss), covariates including socioeconomic condition (education), and social networking (marital status and living alone), results, and conclusions were all collected from the included publications.

### Study risk of bias assessment

Two independent reviewers evaluated the included studies’ risk of bias using the New-castle-Ottawa Scale (NOS) for longitudinal research [25]. This tool uses three groups divided into eight criteria to evaluate each study. First group is the selection of the study sample (representativeness of exposed, selection of non-exposed, ascertainment of exposure, and determining if the outcome was not present at the start). The second group is the comparability (controlling for confounders and additional factors). The last group is relevant to the outcome of interest (assessment of outcomes, follow-up length, adequacy of follow-up). There are three quality levels: good, fair, and poor. For each of the article, the assessment groups are assigned between zero to nine stars, with a higher number indicating higher-quality research. A good quality score is three to four stars for the selection, one to two stars for the comparability, and two to three stars for the result group. Two stars in the selection group, one or two stars in the comparability group, and two or three stars in the outcome group, are considered to be of fair quality. A study is considered to be of poor quality if it receives a score of zero or one in any of the categories of selection, comparability, outcome, or exposure.

To ensure adequate assessment of risk of bias, we also used Risk of Bias in Nonrandomized Studies of Exposures (ROBINS-E) tool [26]. Each study was assessed on seven items of the ROBINS-E: (1) confounding bias, (2) selection bias, (3) exposure bias, (4) departures from intended exposures bias, (5) missing data bias, (6) measurement of outcomes bias, (7) reported result bias. The ROBINS-E questions were answered with options ‘Yes,’ ‘Probably yes,’ ‘Probably no,’ or ‘No.’ before finally judging the risk of bias at study-level and at item-level as ‘low,’ ‘moderate,’ ‘serious,’ or ‘critical’.

### Effect measures

Odds ratio, relative risk, rate ratio, and regression coefficient were used to represent the effect measures for the outcome (tooth loss).

### Synthesis method

Data were gathered from the different studies on exposure (cluster or multiple health-related behaviors), outcome (tooth loss), covariates (socioeconomic condition and social networking), and the effect measure. We also gathered information on follow-up time, sample size, and demographic variables. The review adopted a qualitative synthesis, and individual studies were presented in tables that display the characteristics and the results of the selected papers. It was not possible to do a meta-analysis of the included studies due to their high degree of heterogeneity, notably differences in exposure (health behaviors), measure of outcome (total tooth loss, partial tooth loss), covariate, sample sizes, and follow-up times.

### Report bias of assessment

risk of bias was assessed according to the New-Castle-Ottawa Scale (NOS) for longitudinal research [25] which looked for the outcome and the adequacy of follow-up time and ROBINS-E tool [26] which assessed different sources of bias, such as confounders, exposures, and selection measurements bias. The bias of unpublished research was assessed by searching for grey literature for unpublished results.

### Certainty assessment

Confidence intervals was reported for all the studies.

## Results

### Study selection

**Figure 1:**
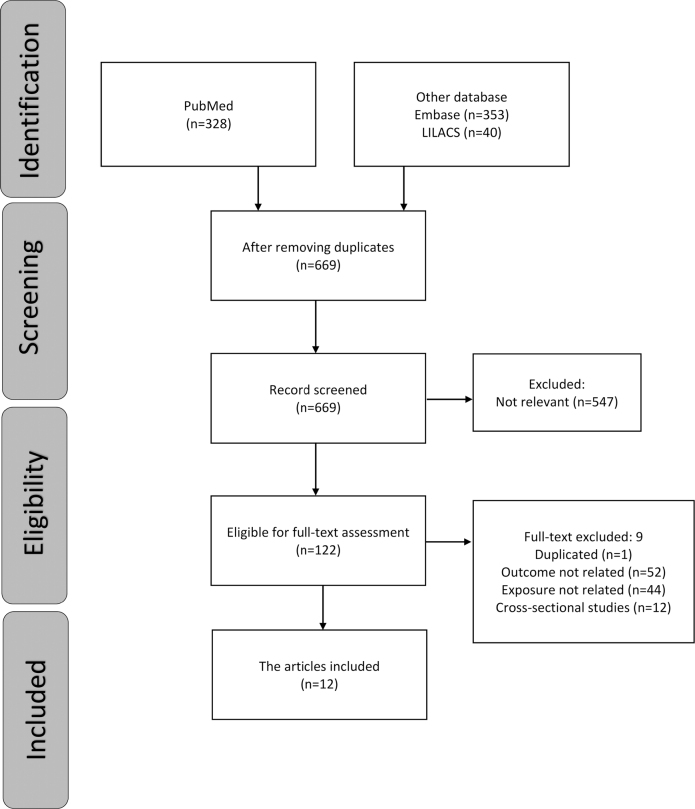
Flowchart of the selection of studies for the review.

The study selection summary is shown in Figure 1. A total of 721 references were identified from three databases (MEDLINE *via* PubMed, EMBASE *via* Ovid and LILACS *via* BIREME). After duplicates were removed, 669 references were included for the title and abstract screening, and 547 articles were found to be irrelevant. Only 12 of the remaining 122 studies met the inclusion criteria after the full reports were evaluated. The number of studies at each stage of the review and the exclusion criteria are depicted in a flowchart (Figure 1). At the final stage, some related papers were excluded because some of them used a cross-sectional analysis of a longitudinal data, and some used a general assessment of oral health rather than a specific assessment of tooth loss. Table 1 displays the included studies’ methodological evaluations based on (NOS) criteria. Table 2 shows the individual studies risk of bias using ROBINS-E tool.

**Table 1:** Methodological assessment of included studies using the Newcastle-Ottawa scales (NOS) with converting scales.

NOS items	Study design	Representativeness of the exposed	Selection of the non-exposed	Ascertainment of exposure	Change in outcome	Comparability	Assessment of outcome	Duration of follow-up	Adequacy of follow-up	Overall quality assessment
Furuta et al. 2021 [27]	Longitudinal	*	*	*	*	**	*	*	*	Good
Kressin et al. 2003 [28]	Longitudinal		*		*	**	*	*	*	Fair
Astrom et al. 2011 [29]	Longitudinal	*	*		*	**		*	*	Good
Weintraub et al. 2019 [30]	Longitudinal	*	*	*	*	**			*	Poor
Furuta et al. 2022 [31]	Longitudinal	*	*	*	*	*	*	*	*	Good
Silva Junior et al. 2019 [32]	Longitudinal	*	*	*	*	*	*			Poor
Copeland et al. 2004 [33]	Longitudinal		*	*	*	*	*	*		Good
Nilsson et al. 2019 [34]	Longitudinal	*	*		*	**	*	*		Good
Torres et al. 2022 [35]	Longitudinal	*	*	*	*	**	*	*	*	Good
Astrom et al. 2021 [36]	Longitudinal	*	*		*	**	*		*	Good
Astrom et al. 2023 [37]	Longitudinal	*	*		*	**	*	*		Good
Xiang Qi et al. 2023 [38]	Longitudinal	*	*		*	**		*	*	Good

**Table 2:** ROBINS-E risk of bias assessment.

Study	Confounding	Exposures	Selection	Post exposure	Missing data	Measurement of outcomes	Reported results	Overall
Furuta at el, 2021 [27]	Moderate	Low	Low	low	Moderate	Moderate	Low	Moderate
Kressin et al. 2003 [28]	Serious	Low	Low	Low	Low	Low	Low	Serious
Astrom et al. 2011 [29]	Low	Low	Low	Low	Moderate	Moderate	Low	Moderate
Weintraub et al. 2019 [30]	Moderate	Low	Low	Low	Low	Moderate	Low	Moderate
Furuta et al. 2022 [31]	Moderate	Low	Low	Low	Low	Low	Low	Moderate
Silva Junior et al. 2019 [32]	Low	Low	Low	Low	Low	Low	Moderate	Moderate
Copeland et al. 2004 [33]	Low	low	Low	Low	Low	Low	Low	Low
Nilsson et al. 2019 [34]	Low	Low	Low	Low	Low	Low	Low	Low
Torres et al. 2022 [35]	Low	Low	Low	Low	Moderate	Low	Low	Moderate
Astom et al. 2021 [36]	Low	Low	Low	Low	Low	Moderate	Low	Moderate
Astrom et al. 2023 [37]	Low	Low	Low	Low	Low	Moderate	Low	Moderate
Xiang Qi et al. 2023 [38]	Low	Low	Low	Low	Low	Moderate	Low	Moderate

### Study criteria

Table 3 shows the characteristics of the included studies. The studies included adults aged 40 and above except for two studies which included participants who were in their 20s at the baseline [28,32]. Most studies had a follow-up period of 10 years and more except for five which had 4, 5, 6, 7, and 8 years of follow-up [30,32,35,36,38]. Tooth loss was clinically examined in 7 studies [27,28,31–35], and self-reported in 5 studies [29,30,36–38]. Tooth loss was defined as the number of missing teeth in 5 studies [27,28,31,33,38], ratio of teeth lost at the follow- up time to teeth present at base line in 1 study [35], complete tooth loss in 1 study [30], missing one or more teeth in 1 study [32], loss of 3 teeth or more during the follow-up period in 1 study [34], lost any teeth in 1 study [29], and lost many or all teeth in 2 studies [36,37]. The major health-related behaviors included in the studies were smoking, alcohol consumption, oral health habits (toothbrushing, flossing and professional oral hygiene prophylaxis) and dental visits.

**Table 3:** Characteristics of the longitudinal studies on the association of cluster health-related behaviors and tooth loss in adults.

Study	Authors	country	Study design	Study sample	Exposures	Outcomes	Covariates	Main findings
Furuta et al. 2021 [27]	Michiko Furuta,Kenji Takeuchi, ToruTakeshita,Yukie Shibata,Shino Suma,Shinya Kageyama,Mikari Asakawa, Jun Hata,Daigo Yoshida,Yoshihiro Shimazaki, Toshiharu Ninomiya, Yoshihisa Yamashita	Japan, 2021	Longitudinal study (10 years follow-up).	in 2007 (*n* = 2665), 2012 (*n* = 2325) and 2017 (*n* = 2285) aged 40–79 years.	Oral hygiene status (toothbrushing frequency, regular dental visits, removal of dental calculus in the past year), current smoking. Dental caries experience and periodontal condition.	Tooth loss (number of missing teeth calculated by examiner as the number of missing teeth).	Age, sex, job at the baseline.	Number of decayed and filled teeth and increase in mean cAl were associated with increasing the number of missing teeth.
Kressin et al. 2003 [28]	N.R. Kressin, U. Boehmer, M.E. Nunn, and A. Spiro III	USA, 2003	Cross-sectional and longitudinal assessments (data collection up to 13 years, and clinical data covering 26 years.).	736 male participants aged 28–80 years old.	Hygiene behaviors (toothbrushing, dental floss use, annual prophylaxis, and combinations of these behaviors).	Tooth retention (number of missing teeth counted by the clinical examiner as the number of missing teeth).	Age, education, smoking habits.	Use of multiple hygiene behaviors was associated with greater tooth retention, cross-sectionally and longitudinally.
Astrom et al. 2011 [29]	Anne N. Astrom, Gunnar Ekback, Sven Ordell, Lennart Unell	Sweden, 2011	Longitudinal study (5, 10 and 15 years Follow-up).	6346 subjects at baseline, 4143 subjects at follow-up between ages 50 and 65	Oral disease and socio-behavioral factors (country of birth, place of living, level of education, marital status, rating of general health status, smoking, regular dental attendance and avoiding preventive dental treatment because of cost, dental pain)	Tooth loss (loss of any teeth self-reported as the loss of any teeth).	Sex, education.	Oral disease-related factors and socio-behavioral characteristics were major risk factors for having tooth loss.
Weintraub et al. 2019 [30]	Jane A. Weintraub, Brian Orleans, Margherita Fontana, Ceib Phillips, and Judith A. Jones	USA, 2019	Longitudinal study (6 years of follow up).	9982 aged 50 years and older	Dental utilization and healthy lifestyle factors (smoking, alcohol drinking, BMI).	Edentulism (complete tooth loss self-reported as being edentulous).	Comorbidities and Functional Status. Demographic variables (age, race/ethnicity, marital status, education levels, wealth).	Non-regular dental attenders and smokers were significantly associated with becoming edentulous.
Furuta et al. 2022 [31]	Michiko Furuta, Kenji Takeuchi, Toru Takeshita, Yukie Shibata, Shino Suma, Shinya Kageyama, Mikari, Asakawa, Yoshihiro Shimazaki, Jun Hata Toshiharu Ninomiya Yoshihisa Yamashita	Japan, 2022	Longitudinal study (10 years of follow up).	1466 participants aged 40 to 79 years.	Regular dental visit, periodontal treatment, tooth brushing, smoking, obesity, and hypertension as categorical variables, triglyceride and HDL cholesterol as continuous variables.	Tooth loss (the number of missing teeth counted by examiner as the number of missing teeth).	Periodontitis and number of DFT as mediator Age, sex, occupational status diabetes, and number of present teeth as covariant.	Baseline periodontitis, dental caries experience, no regular dental visit, periodontal treatment, smoking, and obesity were associated with tooth loss after adjusting for covariates.
Silva Junior et al. 2019 [32]	Manoelito Ferreira Silva Junior, Mar ılia Jesus Batista, Maria da Luz Rosa rio de Sousa	Brazil, 2019	Longitudinal study (4 years of follow up)	240 adults aged 20–64 years.	Type of dental service, service evaluation, marital status, sex, family income, education level, social class, behaviors in oral health including personal health practices (dental flossing, smoking), and dental service utilization.	Tooth loss (missing one or more teeth counted by examiner as missing one or more teeth).	Age, skin color.	The risk factors for tooth loss were: reason for seeking dental services by pain, previous tooth loss and dental caries.
Copeland et al. 2004 [33]	Lynn B. Copeland, Elizabeth A. Krall, L. Jackson Brown, Raul. Garcia, Charles F. Streckfus	USA, 2004	Longitudinal study (10 years of follow up).	94 participants ages 30 to 69 years from (BLSA) compared to 481 men in the same age from (VADLS).	Number of teeth with restorations, number of teeth with caries, probing pocket depth, gingival index, alcohol use, smoking status.	Tooth loss (the number of missing teeth counted by examiner as the number of missing teeth).	Age, sex.	Significant predictors of tooth loss were: percent of teeth with restorations, mean probing pocket depth score, age, tobacco use, alcohol consumption, number of teeth present, and male sex.
Nilsson et al. 2019 [34]	Helena Nilsson, Johan Sanmartin Berglund, Stefan Renvert	Sweden 2019	Longitudinal study (12 years)	375 individual aged 60 and above.	Periodontitis, and dental caries.	Tooth loss (counted by examiner as loss of three or more teeth during the follow up period).).	Age, gender, level of education, use of interdental care devices, frequency of dental visits, living alone, diabetes, MMSE, ischemic heart disease, BMI.	Periodontitis was the strongest predictor of losing more than 3 teeth.
Torres et al. 2022 [35]	Luísa Helena do Nascimento Tôrres, Juliana Balbinot Hilgert, Fernando Neves Hugo, Maria da Luz Rosário de Sousa, Renato José De Marchi	Brazil 2022	Longitudinal study (8 years)	In 2004, 388 aged 60 years or more. In 2012, the follow-up consisted of 199.	Prevalence of caries, GBI, use of partial removable prosthesis, and low stimulated saliva.	Tooth loss (counted by examiner as the ratio of teeth lost at the follow up time to teeth present at the base line).	Age, sex, home location, marital status, schooling, monthly income, and oral health behaviors (smoking, frequency of dental visits, and frequency of tooth brushing.	Tooth loss was linked to factors including age, place of residence, income, marital status, gingival bleeding, and usage of removable partial denture.
Astom et al. 2021 [36]	Anne Nordrehaug Åstrøm, Stein Atle Lie, Ferda O€zkaya	Norway and Sweden 2021	Longitudinal study (5 years)	2947 Norwegian and 4862 Swedish from 65 to 70 years old.	Education.	Tooth loss (self-reported as missing many teeth and edentate)	Smoking, frequency of dental attendance, use of fluoridated toothpaste, alcohol drinking, attitudinal beliefs (as mediators). Social network as confounder.	Education-related inequality in tooth loss was greater in the Norwegian cohort than in the Swedish cohort. this gradient was partially explained by oral behaviors and attitude beliefs.
Astrom et al. 2023 [37]	Anne Nordrehaug Åstrøm, Berit Mastrovito, Josefine Sannevik, Stein Atle Lie	Sweden 2023	Longitudinal study (25 years)	6346 participants aged 50 years old.	Dental attendance, smoking, marital status,	Tooth loss (self-reported as missing many teeth and edentate).	Sex, educational level, country of birth.	Socio-demographic disparities in tooth loss and tooth dissatisfaction persisted throughout the Swedish participants’ middle-aged and older life.
Xiang Qi et al. 2023 [38]	Xiang Qi, Yaolin Pei, Katherine Wang, Shuyu Han, Bei Wu	China 2023	Longitudinal study (7 years)	4268 participants aged 65 years old and more.	Social isolation and loneliness.	Tooth loss (self-reported as the number of remaining teeth)	Lifestyle, oral hygiene behavior, physical and cognitive health as mediators Age, sex, area of residence, education, financial sufficiency as confounders.	Social isolation was significantly associated with fewer remaining teeth and accelerated tooth loss among Chinese older adults.

### Risk of bias in the included studies

According to the Newcastle-Ottawa Scale (NOS), nine of the included papers were considered good quality (scored 9,7,6) [27,29,31,33–38], two poor quality (scored 6 with low score in the outcome section) [30,32], and one fair quality (scored 7 with 2 stars in the selection group) [28]. The differences in the score were because of differences in the selection domain which requires 3 or 4 stars to be considered good quality while the poor score indicates having 0 or 1 star in the outcome domain.

When the risk of bias was assessed by ROBINS-E, nine studies were judged as moderate risk of bias [27,29–32,35–38], two studies were at low risk of bias [33,34] and one study had a serious risk of bias [28]. All studies were at lower risk of exposure bias, selection bias, and post exposure bias. One study was at serious risk of confounding bias [28], three studies were at moderate risk [27,30,31], and the rest were at lower risk of confounding bias. Missing data bias was at a moderate level in three studies [27,29,35] and at a lower level in the other studies. Six studies had a moderate risk of measurement of outcome bias [27,29,30,36–38] while only one study had a moderate risk of reporting results bias [32].

### Results of individual studies

Table 4 presents the association between cluster of/multiple health-related behaviors and tooth loss. Only one study used cluster analysis [28] while the remaining papers assessed multiple behaviors separately. There were seven studies that examined the relationship between tooth loss and oral hygiene practices including toothbrushing frequency [27,28,31,34,35,38], flossing and professional oral hygiene prophylaxis [28,32,34]. Dental visit frequency was reported in nine studies [27,29–32,34–37]. Nine studies examined the association between smoking and tooth loss [28–31,33,35–38], while alcohol consumption was only assessed in four studies [30,33,36,38].

**Table 4:** Association between cluster of health-related behavior and tooth loss.

Study	Independent predictor	Predictor	Description of the predictor	Adjusted measure of association (95%CI)	Covariates	Result	Comments
Furuta et al. 2021 [27]	Tooth loss (calculated by examiner as the number of missing teeth).	Oral health behaviors, periodontal condition, and dental caries.	The frequency of tooth brushing (one time per day, less, two times per day, more) dental visits, (who did, or did not) smoking status: (current smokers, former, non-smokers) Occupational status (clerical support workers, homemaker, unemployed or retired, other jobs). Periodontal examination was performed on all teeth except the third molars at two sites (mesiobuccal and midbuccal). The total number of decayed and filled teeth was used as a measure of assessing dental caries.	After adjustment Rate Ratio: Mean CAL and PPD (RR: 1.01, 95% CI: 1.03–1.07). Numbers of decayed and filled teeth (RR: 1.04, 95% CI: 1.03–1.04).	Age, sex and baseline number of present teeth and job.	Increase in mean CAL and number of decayed and filled teeth were associated with increasing the number of missing teeth. No significant associations with changes in dental plaque, toothbrushing frequency or no regular dental visits over time.	Fewer people participated at the follow-up, which may mean that the population as a whole isn’t as well represented.
Kressin et al. 2003 [28]	Tooth loss (counted by the clinical examiner as the number of missing teeth).	Brushing, floss and prophylaxis use, cluster of behavior.	Toothbrushing was summed to create a toothbrushing score (range, 0–3). Frequency of dental floss use and professional were dichotomized into none vs. any use of dental floss/prophylaxis. Clustering variables: (1) individuals who brushed at least once daily, (2) individuals who brushed at least once daily and received annual prophylaxis but did not floss; (3) individuals who brushed at least once daily and flossed but did not receive annual prophylaxis; (4) individuals who performed all threerecommended behaviors; and (5) those who did none of the behaviors. Education as the highest grade completed, smoking habits were (no smoking, light smokers (half a pack of cigarettes per day), and heavy smokers (a pack of cigarettes or more per day).	Relative Risk: Heavy smokers (RR: 1,90 95% CI 1.30, 2.79) light smokers (RR: 1.51, 95% CI 1.15, 2.00). Education: some college had (RR: 0.66, 95% CI 0.52, 0.84) college degree (RR: 0.56, 95% CI 0.42, 0.74). Brushing more than once a day (RR; 0.62). Baseline flossing (RR: 0.68). Greatest reduction in tooth loss occurring when all three practices were reported (RR: 0.63).	Age, education, smoking habits.	Consistently practicing preventive behaviors over the long term confers greater benefits for tooth retention.	Because only middle-aged and older men were included in the sample, the generalizability of these results is constrained.
Astrom et al. 2011 [29]	Tooth loss (self-reported as the loss of any teeth).	Oral disease and socio-behavioral factors (country of birth, place of living, level of education, civil status, rating of general health status, smoking, regular dental attendance and avoiding preventive dental treatment because of cost, dental pain).	Smoking: (0) no smoking ⁄ on very rare occasions and (1) daily. Toothache: (0) don’t remember any tooth- ache and (1) remember toothache. Country of birth: Sweden (0), Nordic countries (1) others (2). Education: lower (0), high (1), university (2). Civil status: married (0), single (1). Self-reported health: healthy (0), rather healthy (1), rather not healthy (2). Dental attendance: no (0), yes (1). Refrain dental care: no (0), yea (1). Dental pain: during the last 3 months (1), during last year (2), never (3). The predictor variables assessed between 1992 and 2007 were grouped into a conceptual framework according to the life-course stage at which they would be expected to operate as risk factors (early childhood, young adult life, middle-age and early-older-age).	After adjustment Odd Ratio: Avoided dental care because of cost (OR 2.2, 95% CI 1.3–4.0). Being a persistent smoker across time (OR 1.9, 95% CI 1.2– 2.0). Country of birth (OR 1.8, 95% CI 1.1–3.3). Education (OR 1.4, 95% CI 1.1–1.8). Civil status (OR 1.4, 95% CI 1.0–1.9). Reporting pain consistently across time (OR 3.1, 95% CI 2.3–4.0).	Sex and country of birth.	Oral disease-related factors and socio-behavioral characteristics such as refraining from dental care because of financial limitations, acting at earlier and later life-course stages were major risk factors for tooth loss.	The rate of nonresponse, which was approximately 30% in 1992, may suggest that the findings are not inferentially representative of the entire population.
Weintraub et al. 2019 [30]	Tooth loss (self-reported as being edentulous).	Dental utilization, smoking, alcohol drinking, BMI	Regular attender at least once during all three time periods in the study or was a nonregular attender. Smoking status as never smoked, past smoker, and current smoker. Alcohol status was reporting ‘yes’. (BMI) and change in BMI over study period were calculated from self- reported measures. Employment: working all, some, or none of the study period.	After adjustment Odd Ratio: Nonregular dental attenders (OR = 2.74; 95% CI = 2.12–3.53). Current smokers (OR = 2.46; 95% CI = 1.74–3.46). Alcohol drinker (OR = 0.75; 95% CI = 0.60–0.92). BMI (OR = 0.97; 95% CI = 0.94–0.99).	Poor health outcomes and functional status. Age, race/ethnicity, marital status, education levels, wealth, sex, and urban/rural status.	Non-regular dental attenders and smokers were significantly associated with becoming edentulous. Alcohol intake was a protective factor for edentulism. No significant association between marital status and tooth loss.	Social networking was included as marital status and the study did not account for other variables.
Furuta et al. 2022 [31]	Tooth loss (counted by examiner as the number of missing teeth).	Smoking habits, occupational status, tooth brushing frequency, regular dental visits, periodontal treatment. Periodontal condition, Dental caries. Blood pressure, cholesterol and HDL , BMI, diabetes.	Smoking: (current smoker, former smoker and never smoked). Occupational status: (clerical support workers, homemaker, unemployed or retired, and other jobs). The frequency of tooth brushing: (once per day or less, and twice per day or more). Regularly visit: (who did or did not at least once a year). Periodontal treatment: (periodontal treatment within 1 year/over 1 year ago versus no/do not know) (DFT) was used to measure dental caries. Assessment of probing pocket depth (PPD) and clinical attachment level (CAL) of all the teeth (except the third molars), at two sites (mesiobuccal and mid-buccal). Blood samples were collected after overnight fasting.	After full adjustment Odd Ratio: No regular dental visit (OR 1.63, 95% CI 1.13–2.35). Current smoking (OR 1.69, 95% CI 1.10–2.60). Periodontal treatment (OR 2.06, 95% CI 1.47–2.87). Obesity (OR 1.66, 95% CI 1.17–2.35).	Age, sex, occupational status, diabetes, and number of present teeth.	Baseline periodontitis, dental caries experience, no regular dental visit, periodontal treatment, smoking, and obesity were associated with tooth loss after adjusting for covariates. No significant association between toothbrushing and tooth loss. No significant association between DMFT and tooth loss.	This study’s follow-up rate was 60% because older participants were less likely to have a dental exam in the follow-up 2017.
Silva Junior et al. 2019 [32]	Tooth loss (counted by examiner as missing one or more teeth).	Behaviors in oral health, personal health practices, and dental service utilization. Oral health outcomes. Social class, marital status.	Dental flossing (usual or unusual), smoking (yes or no), time since last visit to the dentist (<1 year, 1–2 years, and >2 years), reason for seeking dental services (routine, need, and pain), and frequency of dental visits (regularly or non-regular), type of dental service (public, private, or insurance), service evaluation (good or regular/bad). Presence of visible dental biofilm considered ‘yes’ or ‘no’. Previous tooth loss (more than 5 tooth loss and 4 or less tooth loss); Decayed teeth ‘yes’ or ‘no’. size of the periodontal pocket ‘yes’ (Code 3 and 4 of the index). Social class was assessed according to the classification of Graciano et al. Marital status (stable relationship or no stable relationship), Family income as multiples of the minimum wage (MW): high, medium or low) and Education level (<4 years, between 5–10 years, and >11 years).	Crude model Relative Risk: Frequency of dental visits: (RR −1.73; 95% CI : 1.08–277). After adjustment Relative Risk: Reason for seeking dental services by pain (RR = 2.72; 95.0% CI : 1.04–7.37). Previous tooth loss (RR = 3.01; 95.0% : 1.18–7.73). Decayed teeth (RR = 2.87; 95.0% : 1.22–6.73).	Age, and skin color.	The risk factors for tooth loss were: reason for seeking dental services by pain, previous tooth loss and dental caries. No significant association between dental flossing and tooth loss. No significant association between marital status and tooth loss.	Loss of follow-up was present in this study. Behaviors were only included in the binary analysis and was not included in the adjusted model. The sample size was small (240 participants).
Copeland et al. 2004 [33]	Tooth loss (counted by examiner as the number of missing teeth).	Alcohol use, smoking status, initial number of teeth, number of teeth with restorations, number of teeth with caries, probing pocket depth, gingival index.	Alcohol consumption and tobacco smoking status (questionnaire). DMFT, Periodontal probing pocket depths and gingival assessments were obtained on six index teeth (mesial and buccal sites and the deepest site per tooth).	Regression coefficient (r): Smoking status: (*r* = +0.56, p < .000l). Alcohol intake: (*r* = –1.30, p < .000l). Mean pocket depth: (*r* = +0.59, p < .000l). Teeth with restoration: (*r* = +0.01, p < .000l). Initial number of teeth: (*r* = –0.03, p <.000l).	Sex, age	Among both population the significant predictors of tooth loss were baseline values of percent of teeth with restorations, mean probing pocket depth score, age, tobacco use, alcohol consumption, number of teeth present, and male sex. Alcohol consumption was significantly related to tooth loss rate with a negative effect on (VADL S) and positive effect on (BLSA).	The risk factors for tooth loss in both population was different.
Nilsson et al. 2019 [34]	Tooth loss (counted by examiner as loss of three or more teeth during the follow up period).	Periodontitis and dental caries.	Periodontitis (probing depth), dental caries (an open manifest lesion on the buccal and lingual surface). Utilization of dental healthcare services (at least once a year or less common). Dental hygiene habits toothbrushing frequency and use of interdental care devices. Living conditions (living alone or living with someone). MMSE (cut‐off score <28). diabetes (self‐reported). financial problems (Yes or no).	After adjustment Odds Ratio: Living alone: (OR 2.0; 95% CI: 1.1–3.5). Periodontitis: 2.9; 95%CI : 1.8–4.9).	Dental hygiene, dental visits. Living alone, diabetes, MMSE, ischemic heart disease, BMI, age, gender, level of education.	periodontitis is associated with tooth loss over a 12‐year follow‐up period among older adults. No association between dental visits and tooth loss. No association between the use of interdental devices and tooth loss.	Behaviors were not included in the multivariate model, and it was only reported in the univariate model. Small number of participants (375). No report on smoking and toothbrushing frequency.
Torres et al. 2022 [35]	Tooth loss (counted by examiner as the ratio of teeth lost at the follow up time to teeth present at the base line).	Prevalence of caries, GBI, use of partial removable prosthesis, and low stimulated saliva.	Smoking (smoked more than 100 cigarettes in life), frequency of dental visits (never/occasionally/problem-oriented or regularly) and frequency of tooth brushing (<once a day or ≥ once a day), location (urban/rural), marital status [married or non-married], schooling (<4 years/≥4 years), income (National Minimum Wage), Prevalence of caries at baseline (*D* > 0), GBI at baseline (percentage of sites bleeding after probing the gingival margin), use of partial removable prosthesis, and low stimulated saliva flow at baseline [>1.0 mL/min, saliva was collected].	After adjustment Incidence Rate Ratio (IRR): Being old: (IOR = 1.03; 95% CI: 1.00–1.06). Living in rural area: (IOR = 1.56: 95% CI: 1.17–2.07). Earning wages: (IOR = 1.46: 95% CI: 1.09–1.96). Living alone: (IOR = 1.36: 95%CI : 1.00–1.85). Gingival bleeding: (IOR = 1.01: 95% CI: 1.00–1.02) Prosthesis: (IOR = 2.82: 95% CI: 2.15–3.71).	Oral health behaviors (smoking, dental visits, and frequency of tooth brushing), age, sex, home location, marital status, schooling, and income. No significant association between oral behaviors (toothbrushing, dental visits, and smoking) and tooth loss.	Age, residence location, income, marital status, gingival bleeding, and the use of a removable partial denture were all found to be associated with tooth loss after 8 years of follow-up among older Brazilians.	Loss of follow-up 51%. Small sample size (199 participant at the follow-up)
Astrom et al. 2021 [36]	Tooth loss (self-reported as missing many teeth and edentate).	Education	Education, used as an indicator of early life course social and recoded into (0) higher education, (1) lower education, and other category (5). Social network recorded into (1) weak social ties and (0) strong social ties. Smoking recorded into (0) no smoking, and (1) smoking. Frequency of dental attendance recorded as (0) Attendance at least once a year, (1) less than once a year. Use of fluoridated toothpaste recorded into (0) at least twice daily and (1) daily or less than daily. Toothbrushing recorded into (0) ≤ once daily, and (1) T least twice daily. Use of alcohol recorded into (1) several times a week, and (0) more seldom. Attitudinal beliefs recorded into (0) yes, and (1) no.	In full adjusted model Odd ratio (OR): In Swedish cohort: Smoking: (OR = 0.6: 95% CI: 0.5- 0.6). Alcohol: (OR = 1.2: 95% CI: 1.0- 1.3). Dental visits: (OR = 0.9: 95% CI: 0.8- 1.1). In Norwegian cohort: Smoking: (OR = 0.5: 95% CI: 0.4- 0.6). Alcohol: (OR = 1.3: 95% CI: 1.1- 1.5). Dental visits: (OR = 1.2: 95% CI: 1.0- 1.4).	The study revealed that smoking, more seldom use of alcohol, poor oral hygiene, irregular dental attendance were associated consistently with tooth loss and education across the two cohorts and survey years. Adjusting for oral health behaviors attenuated the education gradients in tooth loss considerably, more strongly so in the Norwegian than in the Swedish cohort. No significant association with brushing and the use of fluoridated toothpaste.	Smoking, frequency of dental attendance, use of fluoridated toothpaste, alcohol drinking, attitudinal beliefs (as mediators). Social network (as confounder).	Because only one social exposure variable (education) was examined, the causes of social disparities in tooth loss was not clear.
Astrom et al. 2023 [37]	Tooth loss (self-reported as missing many teeth and edentate).	Dental attendance, smoking, marital status.	Dental attendance (at least once a year vs less than annually), smoking status (smoking, quitted and no smoking) and perceived health (assessed as (1) perceived to be healthy, and (0) perceived to be not healthy), marital status (married vs nonmarried). Sex (male and female), educational level (low, medium and high) and country of birth (native and foreign borne).	After adjustment Odds Ratio (OR): Dental attendance: (OR = 2.30: 95% CI: 1.92–2.76). Smoking: (OR = 6.01: 95% CI: 4.80–7.54). Education: (OR = 0.13: 95% CI: 0.10–0.17). Marital status: (OR = 1.29: 95% CI: 1.09–1.53).	This study found that socio-demographic disparities in tooth loss and tooth dissatisfaction persisted throughout the Swedish participants’ middle-aged and older life spans. At both the population average and participant-specific levels, these associations were found to hold up after taking into account any potential confounders.	Sex, educational level, country of birth.	The dropouts came from socially disadvantaged groups, which suggests that this study underestimated the gap in oral health between advantaged and disadvantaged groups.
Xiang Qi et al. 2023 [38]	Tooth loss (self-reported as the number of remaining teeth)	Social isolation and loneliness.	Lifestyle was measured as participants’ smoking and drinking status. Oral hygiene behavior was measured as toothbrushing frequency. Social isolation measured as (living alone, not married, lacked social support no social activities). Loneliness was categorized into (0 never lonely, 1 always lonely). Education as (no formal education, with 1– 6 years of schooling, and with more than 6 years of schooling). Physical health was measured as activities of daily living (ADL s) and chronic conditions.	Social isolation: (β= −.05: 95% CI: −0.11, −0.00). Toothbrushing frequency: Less than once a day (β= .12 95% CI: 0.06, 0.18). Once a day (β = .16 95 % CI: 0.10,0.21). More than once a day (β = .17 95%CI: 0.10, 0.24). Education: (β = .10 95% CI: 0.04, 0.15).	Social isolation was associated with fewer teeth and accelerated tooth loss among Chinese older adults. Loneliness was bot associated with fewer teeth nor with the changes in the number of remaining teeth. From all behaviors, only tooth brushing frequency was association with number of remaining teeth.	Lifestyle, oral hygiene behavior, physical and cognitive health as mediators Age, sex, area of residence, education, financial sufficiency as confounders.	Selection bias might result from excluding out participants who dropped out or had passed away.

### Cluster of behaviors

One study used cluster analysis of oral hygiene behaviors to determine whether certain preventive practice combinations had a stronger association with tooth retention than others [28]. The study divided patients into 5 groups ‘(1) people who brush at least once per day; (2) people who brush at least once per day and get annual professional oral hygiene prophylaxis but do not floss; (3) people who flossed and brush their teeth at least once a day but did not get any regular prophylaxis; (4) individuals who engaged in each of the three recommended practices; and (5) those who did none of them.’ There was an almost 49%, 63%, 56%, and 67% reduction in the risk of tooth loss among men who brushed alone, who brushed and received prophylaxis (professional oral hygiene) regularly, who brushed and used floss only, and who brushed, flossed and received regular dental prophylaxis compared to non-regular participant, respectively. In addition to oral hygiene behaviors, the researcher also examined other behavior such as smoking. It was found that persistent smokers (using a pack or more daily) had 1.9 times more likely to lose teeth compared to non-smokers.

### Multiple behaviors

Five other papers included oral hygiene practices along with other behaviors such as smoking [31,34,35] and dental visits [27,31,32,34,35]. In a Japanese study, it was found that frequency of toothbrushing had no association with tooth loss. However, there was a significant association between tooth loss and no regular dental visits when adjusted for periodontal treatment [31]. The study also assessed smoking and found a positive association between being a current smoker and the number of missing teeth. Another study conducted among the same population examined oral hygiene behaviors and dental visits and found no significant associations with the frequency of brushing or dental visits in relation to the number of missing teeth over time [27]. A different study conducted among Swedish population included toothbrushing frequency and smoking but did not report their association with tooth loss [34]. However, the study included other behaviors such as dental visits and the use of interdental devices, but the respective results were not reported in the multivariate model, and they were only reported in the crude model with no association. A Brazilian study examined risk factors for tooth loss and found an association with dental visits, but no association with dental floss in relation to tooth loss [32]. However, these associations were only reported in the crude model. A different study conducted among Brazilian population explored different predictors for tooth loss and found no associations between tooth loss and multiple behaviors including (smoking, access to health services, and toothbrushing frequency) [35].

Two Swedish studies assessed predictors of tooth loss and reported that tooth loss was positively associated with refraining from dental visits [29,37]. The studies also discussed smoking and found that being a current smoker was significantly associated with tooth loss.

Two studies reported alcohol consumption along with other behaviors including dental visits [30] and smoking [30,33]. The first study used data from USA Health Retirement Study examined factors associated with becoming edentulous and reported that being an alcohol drinker has a protective effect on tooth loss compared to being a non-drinker (OR: 0.75) [30]. The same study found that compared to regular dental attenders, non-attendants had nearly three times **the risk of becoming edentulous** [30]. Furthermore, the study also examined smoking and found that current smokers were more likely to become edentulous than individuals who never smoked. The second study conducted among two different USA populations showed that alcohol consumption was associated with tooth loss as a risk factor when the populations combined [33]. However, when comparing the two cohorts separately, the direction of the relation was different showing that alcohol consumption was a risk factor for tooth loss in one population whilst in the other it had a protective effect. The same study also found an association between smoking and tooth loss among both populations.

Two studies discussed multiple behaviors as mediators of the association between social factors and tooth loss [36,38]. The first study demonstrated that multiple behaviors (including smoking, oral hygiene practices, alcohol drinking, and dental visits) were associated with tooth loss and education across the cohorts [36]. The results showed education gradient among Swedish and Norwegian cohort participants who reported tooth loss, with the prevalence of tooth loss higher among those with lower education levels. Adjusting for behaviors attenuated the education gradients in tooth loss especially among Norwegian cohort participants. The second study conducted among Chinese older adults showed that social isolation was significantly associated with fewer remaining teeth, and only tooth brushing frequency was significantly associated with the number of remaining teeth [38]. After the adjustment for mediators including all health behaviors (smoking, alcohol intake, tooth brushing frequency) the association did not change and remained significant.

### Results of syntheses

In general, the review’s findings showed that tooth loss is influenced by multiple behaviors. According to (NOS), nine of the included studies were found to be of good quality [27,29,31,33–38], two had poor quality [30,32], and one had fair quality [28]. According to ROBINS-E risk of bias tool, nine studies were judged as at moderate risk of bias [27,29–32,35–38], one study at serious risk of bias [28], and two studies as at low risk of bias [33,34]. Meta-analysis was not possible because of the high heterogeneity of the included studies regarding differences in exposure (health behaviors), measure of outcome (total tooth loss, partial tooth loss), covariates (socioeconomic factors and social networking), sample sizes, and follow-up times. Only one study used cluster analysis to explore the effect of oral hygiene practices on tooth loss [28], and two studies explored the mediating role of multiple behaviors [36,38].

## Discussion

This systematic review found twelve longitudinal studies from Japan, China, Sweden, Norway, Brazil, and the United States which examined the relationship between cluster of/multiple health-related behaviors and tooth loss. Overall, the research showed evidence of a longitudinal link between cluster of/multiple health-related behaviors and tooth loss.

To our knowledge, no other systematic review assessed the longitudinal relationship between cluster or multiple health-related behaviors and tooth loss. However, there are multiple systematic reviews that examined the association between individual behavior and tooth loss [13,18,20–23,39,40]. It is worth noting that there was unbalance between identified studies that assessed multiple behaviors and studies that used cluster of behaviors. Only one study used cluster analysis to explore the association between oral health behaviors and tooth retention [28], and the rest of the included studies (eleven studies) explored the effect of multiple behavioral factors on tooth loss in the same population. Hence, most of the discussion is relevant to multiple behaviors in the same population.

In this review we found one study suggesting that men who used multiple hygiene practices including brushing, flossing, and regular professional hygiene prophylaxis had better chances of retaining more teeth [28] while four studies found no association between toothbrushing and tooth loss [27,31,35,36]. Another study reported that more frequent tooth brushing was related to higher chances of having more remaining teeth [38]. There are no systematic reviews linking oral hygiene practices and tooth loss. However different reviews have reported that good oral hygiene is related to lower chances of poor oral health outcomes such as dental caries and periodontal diseases [39,40]. One systematic review of observational studies showed that fair and poor oral hygiene practices increase the risk of periodontal diseases [39]. While a Cochrane review stated that the use of interdental devices in addition to toothbrushing may reduce gingival diseases more effectively than toothbrushing alone [40].

Previous systematic reviews and meta-analysis of cross-sectional and longitudinal studies found smoking to be a predictor of tooth loss and suggested that smoking cessation may lower the risk of tooth loss [18,20]. This is similar to our findings that showed a link between smoking and tooth loss in six studies [28–31,36,37]. It was argued that smoking has an impact on the number of teeth through periodontal diseases [41]. While tooth decay is often the main cause of tooth loss, alveolar bone resorption is also an important cause. Hence, nicotine use or cigarette smoking can accelerate alveolar bone loss [42] and smoking cessation may help maintain periodontal and alveolar bone status [43]. Others argued that lower socioeconomic factors impact both smoking and tooth loss, for example, those with lower education levels are more likely to smoke compared to people with higher education levels [44]. This implies that tooth loss among smokers might be a result of their socioeconomic conditions.

In this review, we did not find any study that examined other behaviors and different types of beverages in relation to tooth loss. Only alcohol intake was a risk factor for tooth loss in different studies. However, a recent review of longitudinal and cross-sectional studies investigated the relationship between oral health and beverage consumption and found that tooth loss was cross-sectionally associated with sugary beverages, and coffee consumption [22]. The review also found that alcohol intake can be a protective factor against tooth loss among certain populations (Denmark and Japanese population) [22], a finding consistent with this review. Alcohol intake was found to be a protective factor against tooth loss in two studies among USA population [30,33]. It was argued that consumption of alcohol instead of other beverages, such as sugar-sweetened beverages, could partially explain the observed protective relationship [30]. Sugary drinks can be a risk factor for dental caries and if untreated may lead to tooth loss. Another reason could be the type of alcoholic drinks consumed. Previous evidence reported a relationship between different types of alcoholic drinks and social conditions. Wine drinking was linked to higher socioeconomic status [45] and healthier eating habits [46]. As a result, the current findings might be because of higher socioeconomic status and a healthier lifestyle rather than being the result of alcohol itself [47]. Contradictory, the same systematic review found that alcohol intake can also be a risk factor for tooth loss in other populations (USA, Sweden, Japanese population) [22]. Our review found one paper suggesting that alcohol intake was a risk factor for tooth loss among Swedish and Norwegian populations [36]. As tooth loss is mainly the result of periodontal diseases and tooth decay, it was suggested that drinking can harm the soft tissues by slowing down the salivary flow and accumulating dental plaque which increases the risk of tooth decay and periodontal problems [48]. The flow of saliva aids in the neutralization of acids created by plaque, which helps prevent tooth decay. Lack of saliva enables acids to build up, resulting in gum disease, tooth decay, and periodontal disease [11].

While previous studies linked tooth loss with other behaviors such as diet and physical activity, we did not find any longitudinal study that examined multiple behaviors that included diet and physical activity. However, multiple systematic reviews examined the relationship between fruit and vegetable consumption, as a single behavior, with different oral health outcomes [13,23]. Moreover, a recent systematic review reported an association between physical activity, better oral health (periodontal disease), and oral health behaviors [24].

As behavioral factors are associated with both socioeconomic status and tooth loss, they can be potential mediators of the relationship between oral diseases and social status. The review found two studies that assessed the mediating role of multiple behaviors (including smoking, oral hygiene practices, alcohol drinking, and dental visits) between social factors and tooth loss [36,38]. The studies explained that behavioral factors could explain in some way the social differences in tooth loss. Previous evidence suggested that behavioral and psychological factors could be potential mediators between social factors and dental diseases and could explain the social inequalities in oral health [49–51].

The major impact of tooth loss on oral and general health is a public health concern, especially among older adults. Tooth loss can have a negative impact on the quality of life of patients, change masticatory function, impair nutritional intake, and affect aesthetics [52]. Finding different mechanisms for tooth loss impact on older adults can guide our prevention towards more practical strategies and behavioral changes to reduce the negative influences of tooth loss [2].

This systematic review has some limitations that should be addressed. First, it was not possible to perform a meta-analysis because of variances in behavioral characteristics, the measure of outcome, sample size, and follow-up duration that prevented the pooling of the results. Second, the discussion of this review was limited due to the lack of studies that examined the relationship between clustering of health-related behavior and tooth loss. Third, the limitations of the included studies such as the limited generalizability of the sample, the variations of the follow-up period, and the different methods of collection of data, have impacted the quality of the evidence produced by the review.

The review has some implications. The findings highlight the importance of exploring the aggregate effect of multiple behaviors on tooth loss to help maintain teeth at an older age. Given the fact that multiple behaviors affect other health outcomes, it is necessary to address and target multiple behaviors, particularly those that are not directly linked to tooth loss and may also have other health benefits. Future research on cluster of health-related behavior and tooth loss should investigate explanatory pathways to demonstrate how health-behaviors affect oral health over time and assess the impact of cluster of health behaviors on tooth loss.

## Conclusion

The majority of previous research on tooth loss have examined the effect of multiple health related behavior and not cluster of behaviors. The overall findings of the review imply that multiple health-related behaviors were longitudinally associated with tooth loss in the same population. The included studies varied according to exposures, sample sizes, and follow-up times. Further research is required to explore the different factors that contribute to the association between health-related behaviors and tooth loss.

## Authors’ contributions

F.A. and W.S. contributed to the conceptualization and the methodological design of the review, data curation and interpretation, editing and drafting of the article and approval of the manuscript. W.S. and E.H. contributed to the revision of the manuscript and final approval for publication.

## Disclosure statement

No potential conflict of interest was reported by the author(s).

## Supporting information

The study protocol was registered in the International Prospective Register of Systematic Reviews (PROSPERO) (Registration number CRD42022367174). File S1 include Prisma 2020 checklist. The search strat-egy used is shown in (Appendix 1, supplementary material).

## ORCID

Fatimah Alobaidi http://orcid.org/0009-0001-0590-8291

Ellie Heidari http://orcid.org/0000-0002-6339-9480

Wael Sabbah http://orcid.org/0000-0002-4609-0991

## Supplementary Material

Systematic review of longitudinal studies on the association between cluster of health-related behaviors and tooth loss among adults

## References

[1] PHE. What do we know about the oral health of older people in England and Wales? A review of oral health surveys of older people. London: PHE publications; 2015.

[2] Jiang CM, Chu CH, Duangthip D, et al. Global perspectives of oral health policies and oral healthcare schemes for older adult populations. Front Oral Health. 2021;2:703526. doi:10.3389/froh.2021.703526. 35048040 PMC8757822

[3] Gilbert GH, Miller MK, Duncan RP, et al. Tooth-specific and person-level predictors of 24-month tooth loss among older adults. Community Dent Oral Epidemiol. 1999;27(5):372–385. doi:10.1111/j.1600-0528.1999.tb02034.x. 10503798

[4] Chen X, Clark JJ. Multidimensional risk assessment for tooth loss in a geriatric population with diverse medical and dental backgrounds. J Am Geriatr Soc. 2011;59(6):1116–1122. doi:10.1111/j.1532-5415.2011.03425.x. 21649626

[5] Nakahori N, Sekine M, Yamada M, et al. Socioeconomic status and remaining teeth in Japan: results from the Toyama dementia survey. BMC Public Health. 2019;19(1):691. doi:10.1186/s12889-019-7068-7. 31164111 PMC6549260

[6] Spring B, Moller AC, Coons MJ. Multiple health behaviours: overview and implications. J Public Health. 2012;34(Suppl 1):i3–10. doi:10.1093/pubmed/fdr111. PMC3284863 22363028

[7] Ishikawa S, Konta T, Susa S, et al. Risk factors for tooth loss in community-dwelling Japanese aged 40 years and older: the Yamagata (takahata) study. Clin Oral Investig. 2019;23(4):1753–1760. doi:10.1007/s00784-018-2604-x. 30167794

[8] Similä T, Virtanen JI. Association between smoking intensity and duration and tooth loss among Finnish Middle-aged adults: the Northern Finland birth cohort 1966 project. BMC Public Health. 2015;15(1):1141. doi:10.1186/s12889-015-2450-6. 26576994 PMC4650303

[9] Holm G. Smoking as an additional risk for tooth loss. J Periodontol. 1994;65(11):996–1001. doi:10.1902/jop.1994.65.11.996. 7853136

[10] Hanioka T, Ojima M, Tanaka K, et al. Association of total tooth loss with smoking, drinking alcohol and nutrition in elderly Japanese: analysis of national database. Gerodontology. 2007;24(2):87–92. doi:10.1111/j.1741-2358.2007.00166.x. 17518955

[11] Tezal M, Grossi SG, Ho AW, et al. The effect of alcohol consumption on periodontal disease. J Periodontol. 2001;72(2):183–189. doi:10.1902/jop.2001.72.2.183. 11288791

[12] Sanchez GFL, Smith L, Koyanagi A, et al. Associations between self-reported physical activity and oral health: a cross-sectional analysis in 17,777 Spanish adults. Br Dent J. 2020;228(5):361–365. doi:10.1038/s41415-020-1306-3. 32170257

[13] Skoczek-Rubińska A, Bajerska J, Menclewicz K. Effects of fruit and vegetables intake in periodontal diseases: a systematic review. Dent Med Probl. 2018;55(4):431–439. doi:10.17219/dmp/99072. 30592392

[14] Meader N, King K, Moe-Byrne T, et al. A systematic review on the clustering and co-occurrence of multiple risk behaviours. BMC Public Health. 2016;16(1):657. doi:10.1186/s12889-016-3373-6. 27473458 PMC4966774

[15] Austregésilo SC, de Goes PSA, de Sena Júnior MR, et al. Clustering of oral and general health risk behaviors among adolescents. Prev Med Rep. 2019;15:100936. doi:10.1016/j.pmedr.2019.100936. 31372328 PMC6661380

[16] Simancas-Pallares MA, Ginnis J, Vann WF, Jr., et al. Children’s oral health-related behaviours and early childhood caries: a latent class analysis. Community Dent Oral Epidemiol. 2022;50(3):147–155. doi:10.1111/cdoe.12645. 33987840 PMC8589886

[17] Nouraei N, Sabbah W. Health-risk behaviours co-occur among children with untreated caries. Int J Dent Hyg. 2022;12603. doi:10.1111/idh.12603. 35922901

[18] Souto MLS, Rovai ES, Villar CC, et al. Effect of smoking cessation on tooth loss: a systematic review with meta-analysis. BMC Oral Health. 2019;19(1):245. doi:10.1186/s12903-019-0930-2. 31718636 PMC6852780

[19] Galvin S, Anishchuk S, Healy CM, et al. Smoking, tooth loss and oral hygiene practices have significant and site-specific impacts on the microbiome of oral mucosal surfaces: a cross-sectional study. J Oral Microbiol. 2023;15:2263971. 37795170 10.1080/20002297.2023.2263971 PMC10547447

[20] Vieira TR, Martins CC, Cyrino RM, et al. Effects of smoking on tooth loss among individuals under periodontal maintenance therapy: a systematic review and meta-analysis. Cad Saude Publica. 2018;34:e00024918. 30281706 10.1590/0102-311X00024918

[21] Pulikkotil SJ, Nath S, Dharamarajan L, et al. Alcohol consumption is associated with periodontitis. A systematic review and meta-analysis of observational studies. Community Dent Health. 2020;37:12–21. 32031339 10.1922/CDH_4569Pulikkotil10

[22] Zupo R, Castellana F, De Nucci S, et al. Beverages consumption and oral health in the aging population: a systematic review. Front Nutr. 2021;8:762383. doi:10.3389/fnut.2021.762383. 34778347 PMC8579113

[23] Smits KPJ, Listl S, Jevdjevic M. Vegetarian diet and its possible influence on dental health: a systematic literature review. Community Dent Oral Epidemiol. 2020;48(1):7–13. doi:10.1111/cdoe.12498. 31571246 PMC6972589

[24] Medapati A, Pachava S. Effect of physical activity on oral health: a systematic review. J Indian Assoc Public Health Dent. 2022;20(2):125–130. doi:10.4103/jiaphd.jiaphd_142_21.

[25] Wells GA, Shea B, O’Connell D, et al. The Newcastle-Ottawa Scale (NOS) for assessing the quality of nonrandomised studies in meta-analyses. 2014.

[26] Morgan RL, Thayer KA, Santesso N, et al. A risk of bias instrument for non-randomized studies of exposures: a users’ guide to its application in the context of GRADE. Environ Int. 2019;122:168–184. doi:10.1016/j.envint.2018.11.004. 30473382 PMC8221004

[27] Furuta M, Takeuchi K, Takeshita T, et al. 10-year trend of tooth loss and associated factors in a japanese population-based longitudinal study. BMJ Open. 2021;11(8):e048114. doi:10.1136/bmjopen-2020-048114. PMC8375749 34408043

[28] Kressin NR, Boehmer U, Nunn ME, et al. Increased preventive practices lead to greater tooth retention. J Dent Res. 2003;82(3):223–227. doi:10.1177/154405910308200314. 12598553

[29] Åstrøm AN, Ekback G, Ordell S, et al. Socio-behavioral predictors of changes in dentition status: a prospective analysis of the 1942 swedish birth cohort. Community Dent Oral Epidemiol. 2011;39(4):300–310. doi:10.1111/j.1600-0528.2010.00594.x. 21114515

[30] Weintraub JA, Orleans B, Fontana M, et al. Factors associated with becoming edentulous in the US health and retirement study. J Am Geriatr Soc. 2019;67(11):2318–2324. doi:10.1111/jgs.16079. 31335967

[31] Furuta M, Takeuchi K, Takeshita T, et al. Baseline periodontal status and modifiable risk factors are associated with tooth loss over a 10-year period: estimates of population attributable risk in a japanese community. J Periodontol. 2022;93(4):526–536. doi:10.1002/JPER.21-0191. 34435683 PMC9305417

[32] Silva Junior MF, Batista MJ, de Sousa M. Risk factors for tooth loss in adults: a population-based prospective cohort study. PLoS One. 2019;14(7):e0219240. doi:10.1371/journal.pone.0219240. 31329623 PMC6645523

[33] Copeland LB, Krall EA, Brown LJ, et al. Predictors of tooth loss in two US adult populations. J Public Health Dent. 2004;64(1):31–37. doi:10.1111/j.1752-7325.2004.tb02723.x. 15078059

[34] Nilsson H, Sanmartin Berglund J, Renvert S. Longitudinal evaluation of periodontitis and tooth loss among older adults. J Clin Periodontol. 2019;46(10):1041–1049. doi:10.1111/jcpe.13167. 31294471

[35] Tôrres L, Hilgert JB, Hugo FN, et al. Predictors of tooth loss in Brazilian older adults: an 8-year follow-up. Gerodontology. 2022;40(2):207–212. doi:10.1111/ger.12634. 35474327

[36] Åstrøm AN, Lie SA, Özkaya F. Influences of behaviour and attitude on education related inequality in tooth loss: findings from Norway and Sweden over 5 years of follow-up. Acta Odontol Scand. 2021;79(2):81–88. doi:10.1080/00016357.2020.1785002. 32584634

[37] Åstrøm AN, Mastrovito B, Sannevik J, et al. Oral health inequalities in Swedish older adults over 25 years of follow-up. Gerodontology. 2023 [cited 2023 March 7]. doi:10.1111/ger.12680. 36880598

[38] Qi X, Pei Y, Wang K, et al. Social isolation, loneliness and accelerated tooth loss among Chinese older adults: a longitudinal study. Community Dent Oral Epidemiol. 2023;51(2):201–210. doi:10.1111/cdoe.12727. 35040179 PMC9288561

[39] Lertpimonchai A, Rattanasiri S, Arj-Ong Vallibhakara S, et al. The association between oral hygiene and periodontitis: a systematic review and meta-analysis. Int Dent J. 2017;67(6):332–343. doi:10.1111/idj.12317. 28646499 PMC5724709

[40] Worthington HV, MacDonald L, Poklepovic Pericic T, et al. Home use of interdental cleaning devices, in addition to toothbrushing, for preventing and controlling periodontal diseases and dental caries. Cochrane Database Syst Rev. 2019;4:Cd012018. 30968949 10.1002/14651858.CD012018.pub2 PMC6953268

[41] Burt BA, Ismail AI, Morrison EC, et al. Risk factors for tooth loss over a 28-year period. J Dent Res. 1990;69(5):1126–1130. doi:10.1177/00220345900690050201. 2335645

[42] Kubota M, Yanagita M, Mori K, et al. The effects of cigarette smoke condensate and nicotine on periodontal tissue in a periodontitis model mouse. PLoS One. 2016;11(5):e0155594. doi:10.1371/journal.pone.0155594. 27203240 PMC4874667

[43] Tonetti MS. Cigarette smoking and periodontal diseases: etiology and management of disease. Ann Periodontol. 1998;3(1):88–101. doi:10.1902/annals.1998.3.1.88. 9722693

[44] Buchwald S, Kocher T, Biffar R, et al. Tooth loss and periodontitis by socio-economic status and inflammation in a longitudinal population-based study. J Clin Periodontol. 2013;40(3):203–211. doi:10.1111/jcpe.12056. 23379538

[45] Mortensen EL, Jensen HH, Sanders SA, et al. Better psychological functioning and higher social status may largely explain the apparent health benefits of wine: a study of wine and beer drinking in young Danish adults. Arch Intern Med. 2001;161(15):1844–1848. doi:10.1001/archinte.161.15.1844. 11493125

[46] Tjønneland A, Grønbaek M, Stripp C, et al. Wine intake and diet in a random sample of 48763 danish men and women. Am J Clin Nutr. 1999;69(1):49–54. doi:10.1093/ajcn/69.1.49. 9925122

[47] Heegaard K, Avlund K, Holm-Pedersen P, et al. Amount and type of alcohol consumption and missing teeth among community-dwelling older adults: findings from the Copenhagen oral health senior study. J Public Health Dent. 2011;71(4):318–326. doi:10.1111/j.1752-7325.2011.00276.x. 22320290

[48] Dukić W, Dobrijević TT, Katunarić M, et al. Caries prevalence in chronic alcoholics and the relationship to salivary flow rate and pH. Cent Eur J Public Health. 2013;21(1):43–47. doi:10.21101/cejph.a3796. 23741900

[49] Celeste RK, Gonçalves LG, Faerstein E, et al. The role of potential mediators in racial inequalities in tooth loss: the Pró-Saúde study. Community Dent Oral Epidemiol. 2013;41(6):509–516. doi:10.1111/cdoe.12051. 23647615

[50] Sabbah W, Suominen AL, Vehkalahti MM, et al. The role of behaviour in inequality in increments of dental caries among finnish adults. Caries Res. 2015;49(1):34–40. doi:10.1159/000366491. 25401295

[51] Bernabé E, Suominen AL, Nordblad A, et al. Education level and oral health in Finnish adults: evidence from different lifecourse models. J Clin Periodontol. 2011;38(1):25–32. doi: 10.1111/j.1600-051X.2010.01647.x. 21058971

[52] Xu K, Yu W, Li Y, et al. Association between tooth loss and hyper-tension: a systematic review and meta-analysis. J Dent. 2022;123:104178. doi:10.1016/j.jdent.2022.104178. 35661800

