# Supplementary material for: Systematic review of longitudinal studies on the association between cluster of health-related behaviors and tooth loss among adults
Source: Acta Odontol Scand. 2024 Apr 3;83:40284. doi: 10.1080/00016357.2023.2287718 (PMC11302646; doi:10.1080/00016357.2023.2287718)
Supplement: Systematic review of longitudinal studies on the association between cluster of health-related behaviors and tooth loss among adults [file AOS-83-40284-s1.pdf]

Supplementary material has been published as submitted. It has not been copyedited or typeset by Acta Odontologica Scandinavica.

Search History/

1/PubMed:
((edentulous) AND (behaviour)) AND (Longitudinal)

((tooth loss) AND (behaviour)) AND (Longitudinal)

((oral health) AND (behaviour)) AND (Longitudinal)

((tooth loss[Title/Abstract]) AND (risk factors[Title/Abstract])) AND (longitudinal[Title/Abstract])

((tooth loss[Title/Abstract]) AND (risk behavior[Title/Abstract])) AND (longitudinal[Title/Abstract])

((tooth loss[Title/Abstract]) AND (risk behaviour[Title/Abstract])) AND (longitudinal[Title/Abstract])

((tooth loss[Title/Abstract]) AND (behaviour[Title/Abstract])) AND (longitudinal[Title/Abstract])

((tooth loss[Title/Abstract]) AND (behavior[Title/Abstract])) AND (longitudinal[Title/Abstract])

((tooth retention) AND (behavior)) AND (longitudinal)

((functional dentition) AND (behavior)) AND (longitudinal)

((edentulous) AND (behavior)) AND (Longitudinal)

((edentulous[Title/Abstract]) AND (behavior[Title/Abstract])) AND (Longitudinal[Title/Abstract])

(((oral health) AND (dental)) AND (behaviour[Title/Abstract])) AND (Longitudinal)

(((oral health[Title/Abstract]) OR (dental[Title/Abstract])) AND (cluster of behaviour)) AND (Longitudinal)

((oral health) AND (cluster of behaviour)) AND (Longitudinal)

2/Ovid (Embase):

1 oral health.mp. (34582)
2 tooth loss.mp. or exp periodontal disease/ (113130)
3 absence of teeth.mp. (252)
4 edentulous.mp. or exp edentulous jaw/ or exp edentulousness/ (12832)
5 function dentition.mp. (2)
6 health behavio?r.mp. or exp health behavior/ or exp attitude to health/ (479945)
7 risk factor.mp. or exp risk factor/ (1412259)
8 longitudinal.mp. or exp longitudinal study/ (461109)
9 1 or 2 or 3 or 4 or 5 (149570)
10 6 or 7 (1820354)
11 8 and 9 and 10 (797)
12 limit 11 to (english and (adult <18 to 64 years> or aged <65+ years>) and last 22 years) (353)

3/ LILACS:

tooth loss OR oral health AND behavior AND longitudinal AND (db:("LILACS") AND la:("en")) AND (year_cluster:[2000 TO 2022]) (2)

(oral health) AND (behavior) AND (longitudinal) AND (db:("LILACS") AND la:("en")) AND (year_cluster:[2000 TO 2022]) (14)

(absence of teeth) AND (behavior) AND (longitudinal) AND (db:("LILACS") AND la:("en")) AND (year_cluster:[2000 TO 2022]) (2)

(edentulous) AND (behavior) AND (longitudinal) AND (db:("LILACS") AND la:("en")) AND (year_cluster:[2000 TO 2022]) (0)

(oral health) AND (risk factor) AND (longitudinal) AND (db:("LILACS") AND la:("en")) AND (year_cluster:[2000 TO 2022]) (29)

(tooth loss) AND (risk factor) AND (longitudinal) AND (db:("LILACS") AND la:("en")) AND (year_cluster:[2000 TO 2022]) (3)

(edentulous) AND (risk factor) AND (longitudinal) AND (db:("LILACS") AND la:("en")) AND (year_cluster:[2000 TO 2022]) (0)

(absence of teeth) AND (risk factor) AND (longitudinal) AND (db:("LILACS") AND la:("en")) AND (year_cluster:[2000 TO 2022]) (0)

(function dentition) AND (risk factor) AND (longitudinal) AND (db:("LILACS") AND la:("en")) AND (year_cluster:[2000 TO 2022]) (0)

(function dentition) AND (behavior) AND (longitudinal) AND (db:("LILACS") AND la:("en")) AND (year_cluster:[2000 TO 2022]) (1)
